# Bioinformatic Amplicon Read Processing Strategies Strongly Affect Eukaryotic Diversity and the Taxonomic Composition of Communities

**DOI:** 10.1371/journal.pone.0130035

**Published:** 2015-06-05

**Authors:** Markus Majaneva, Kirsi Hyytiäinen, Sirkka Liisa Varvio, Satoshi Nagai, Jaanika Blomster

**Affiliations:** 1 Department of Environmental Sciences, University of Helsinki, Helsinki, Finland; 2 Tvärminne Zoological Station, University of Helsinki, Hanko, Finland; 3 Department of Mathematics and Statistics, University of Helsinki, Helsinki, Finland; 4 Research Center for Aquatic Genomics, National Research Institute of Fisheries Science, Yokohama, Japan; Graz University of Technology (TU Graz), AUSTRIA

## Abstract

Amplicon read sequencing has revolutionized the field of microbial diversity studies. The technique has been developed for bacterial assemblages and has undergone rigorous testing with mock communities. However, due to the great complexity of eukaryotes and the numbers of different rDNA copies, analyzing eukaryotic diversity is more demanding than analyzing bacterial or mock communities, so studies are needed that test the methods of analyses on taxonomically diverse natural communities. In this study, we used 20 samples collected from the Baltic Sea ice, slush and under-ice water to investigate three program packages (UPARSE, mothur and QIIME) and 18 different bioinformatic strategies implemented in them. Our aim was to assess the impact of the initial steps of bioinformatic strategies on the results when analyzing natural eukaryotic communities. We found significant differences among the strategies in resulting read length, number of OTUs and estimates of diversity as well as clear differences in the taxonomic composition of communities. The differences arose mainly because of the variable number of chimeric reads that passed the pre-processing steps. Singleton removal and denoising substantially lowered the number of errors. Our study showed that the initial steps of the bioinformatic amplicon read processing strategies require careful consideration before applying them to eukaryotic communities.

## Introduction

Historically, the diversity of protists has been determined with laborious morphological surveys [1,2], in which taxon identification requires expertise that is acquired over years of microscopic work. Planktonic protistan communities harbor a large number of species that are easily overlooked or missed in sampling and counting due to very low cell abundance [3–5]. This tendency has led to the underestimation of protistan species richness in examined environments.

This underestimation became more evident with the construction of 18S ribosomal RNA gene clone libraries from environmental samples [6–8]. The clone library studies have revealed novel taxa and greater-than-expected protistan richness. Although one can study larger volumes of water (up to tens of liters) with clone libraries (the sample is collected on a filter from which DNA is extracted and further processed) than with a microscope, the clone library approach and the microscopic approach share a similar limiting factor: the number of observations per sample (sequenced clones) is low, usually only a few hundred, depending on how many clones are picked. Rarefaction analyses show that this approach has far from thoroughly sampled the richness [8].

The emergence of the different next-generation sequencing techniques (454, Illumina, SOLiD, etc.), which can massively sequence 90 to 1000-bp-long DNA fragments, was a step towards a more precise molecular-based assessment of protistan richness in examined environments. One can sequence tens of thousands of sequences (amplicon reads) from a single sample, including rare taxa, and, in theory, estimate the protistan richness of an environment more accurately [9,10].

Bacterial communities were the first subjects of clone libraries, amplicon read sequencing and downstream analyses. The analyses evoked vivid discussion about the so-called rare biosphere that later subsided when more sophisticated amplicon read quality and chimera detection methods revealed that most of the rare biosphere was due to errors in the new sequencing technologies [11]. The errors included, for instance, chimeric reads, reads with indels, and homopolymer miscounts (e.g., TTT is read as TT or TTTT). Artificial (“mock”) community analyses have shown that the number of operational taxonomic units (OTUs) often far exceeds the number of actual species in these communities [12–14].

Several detection methods have been developed to overcome the problem of chimeric reads produced in PCR amplification [15–22]. Of these, Chimera Slayer [20] and UCHIME [22] have proved to be the most sensitive [22]. Chimera Slayer searches a multiple alignment of chimera-free reference sequences. Alternatively, aligned sample reads can serve as a reference. UCHIME can be run against a reference database, but it is not required. However, no method eliminates chimeras entirely [20].

Denoising methods have also been developed to limit the ‘noise’ produced by amplicon read sequencing techniques. These methods can precluster rarer reads (most likely erroneous) with related more abundant reads [23] or produce a cluster consensus read [24]. Alternatively, denoising methods can use raw sequencing data in the form of flowgrams [21,25,26]. Research has shown that denoising eliminates actual OTUs [27,28], and can therefore underestimate diversity.

Assessing eukaryotic diversity with molecular methods is more complicated than it is for Bacteria and Archaea. The number of 18S rRNA gene copies per cell varies from one to tens of thousands among different eukaryotes [29–31], resulting in values that represent not the number of cells but the number of 18S rRNA gene copies in the sample. Also, the variability in the 18S rDNA differs across eukaryotic lineages [32,33], and no universal level of sequence similarity is available. For example, ciliates require a 98% level of similarity for analyzing their diversity at the species level [33], while Behnke et al. [13] showed that in pyrosequenced Rhizaria even a 91% level of similarity will overestimate the species richness. Recently, several studies have addressed the amplicon read overestimation of eukaryotic diversity. However, these studies have concentrated on mock communities [12,13,28,34,35] or on certain taxonomic groups [27,36–40], and their results must be verified for different, taxonomically diverse natural communities.

In this paper, we show that the choice of bioinformatic strategy strongly affects estimates of diversity of Baltic Sea ice and water samples that include members of at least 28 diverse eukaryotic lineages [41]. We used singleton removal, quality control filtering, two different chimera detection methods and two denoising methods to test 18 different strategies implemented in mothur [42], QIIME [43] and USEARCH (or UPARSE) [34]. We also manually validated the chimera detection methods from a subset of samples.

## Material and Methods

### Sampling

We collected 20 samples (15 sea-ice, 3 slush and 2 under-ice water samples) from three R/V Aranda sea-ice cruise stations (Gulf of Finland, Baltic Sea, 8–19 March, 2010): a drift-ice station on 9 March (59°55.67' 26° 01.082'), a heavily packed fast-ice station on 11 March (60°14.30' 26°37.563'), and a level fast-ice station on 13 March (60°19.664' 26°51.730'). The field work required no permits or approvals.

We collected the ice samples with a motorized CRREL-type ice-coring auger (9 cm internal diameter, Kovacs Enterprises). We obtained five ice cores from each station and immediately sectioned them into five pieces of approximately equal size: surface, upper intermediate, middle, lower intermediate and bottom sections. Thus, the sections varied in size, depending on the ice thickness of (43–112 cm) each core. At each location, we placed all five surface sections into a plastic bag, all five bottom sections into another plastic bag, and so on. The ice was then crushed inside the bags, transferred to a bucket and left to melt in darkness at +4°C. We took three replicate slush samples at the fast ice station, shoveled them from an approximately 50 cm x 50 cm square with a hand shovel, and left them to melt in a basket in darkness at +4°C. We sampled the under-ice water by submersing three one-liter bottles in the corer holes at the drift and fast ice stations.

For the DNA extraction, 550–600 mL of water, melted sea-ice and slush was sequentially filtered with 47 mm diameter 180-μm pore-size nylon filters (Millipore), 20 μm Polyvinylidene fluoride filters (Durapore, Millipore), and 0.2-μm mixed cellulose ester membrane filters (Schleicher and Schuell). We stored the 0.2- and 20-μm filters in liquid nitrogen while on-board and transferred them to a -80°C freezer on shore until further processing.

### DNA Extraction, PCR Amplification and Sequencing

We soaked the 0.2-μm filters in DNA lysis buffer (100 mM Tris, 50 mM EDTA, 500 mM NaCl, 0.6% w/v SDS) and extracted total DNA from the filter with the phenol-chloroform method [44].

**Fig 1:**
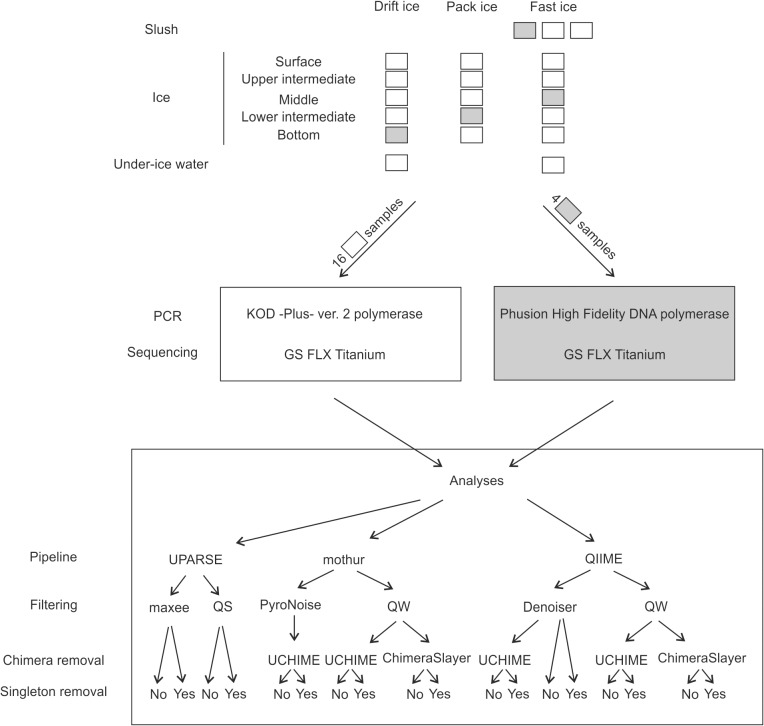
Experimental design. The 20 obtained samples were divided into two sets: 16 samples were amplified and sequenced in Japan, and 4 samples (grey) were amplified and sequenced in Finland. For downstream analyses, we combined and analyzed these two sets with UPARSE, mothur and QIIME program packages. Within the program packages, we used varying quality filtering methods: in UPARSE, maximum expected error (maxee) and quality score (QS) filtering methods; in mothur, PyroNoise denoising and quality score window (QW) filtering; and in QIIME Denoiser denoising and QW filtering. In addition, we tested UCHIME and Chimera Slayer chimera detection methods in mothur and QIIME.

Amplification of the approximately 480-bp long 18S rRNA gene fragment (including the variable sites V7, V8 and V9) took place in two separate laboratories (Fig 1), using primers 18S-F1289 and 18S-R1772 [45] with attached sample-specific 6-bp-long barcode tags. Of the 20 samples, 16 were amplified with KOD-Plus- ver. 2 (TOYOBO Co. Inc., Osaka, Japan) polymerase under the following conditions: initial denaturation at 94°C for 2 min followed by 25 cycles at 94°C for 15 sec, at 50°C for 30 sec and at 68°C for 1 min. Each sample was amplified once. We used a High Pure PCR Product Purification Kit (Roche Diagnostics) to purify and concentrate the PCR reactions. We used Phusion High-Fidelity DNA Polymerase (Thermo Scientific Inc., Waltham, MA, USA) to amplify the four remaining samples under the following conditions: initial denaturation at 98°C for 30 sec followed by 30 cycles at 98°C for 10 sec, at 65°C for 30 sec, and at 72°C for 10 sec, with a final extension at 72°C for 5 min. The PCR took place in two phases: in the first phase, in eight replicates, and in the second phase, in three replicates. We pooled the replicates both between and after the amplifications. We then purified and concentrated the PCR reactions with an AMPure XP (Beckman Coulter Inc., Brea, CA, USA) PCR purification kit.

We mixed the PCR products in equimolar ratio and used a GS FLX Titanium Rapid Library Preparation Kit (Roche) to prepare a DNA library. We then amplified these pooled libraries with beads by emulsion polymerase chain reaction and pyrosequenced the amplified fragments in the DNA libraries on a picotiter plate with 454 GS FLX Titanium system and reagents (Roche) at the Research Center for Aquatic Genomics (Yokohama, Japan; 16 samples) and at the Institute of Biotechnology (Helsinki, Finland; 4 samples).

### Processing of Reads

Three amplicon read processing pipelines (mothur v.1.34.3 [42], QIIME 1.8.0 [43] and UPARSE as implemented in USEARCH v7.0.1090 [34]) were used. The reads were grouped into OTUs at 90, 95, 96, 97, 98, 99 and 100-% similarity levels. OTUs occurring only once (singletons) were either retained or removed from the dataset (Fig 1). In mothur (S1 Text), we followed the Schloss SOP pipeline [14] in www.mothur.org/wiki/ (accessed 17 January 2014). We tested both the shhh.flows quality filtering, which utilizes the PyroNoise flowgram denoising algorithm [25], and the trim.seqs command (quality window method, QW), which cuts reads when their average quality score over a 50-bp window drops below 25 (35 in default SOP). With both quality filtering methods, we also eliminated reads with > 6 homopolymers (> 8 in default), reads with ambiguous bases, reads with > zero mismatches in the barcode (> 1 in default) and the primer (> 2 in default) sequence. We aligned the unique reads against the recreated SILVA SEED database v119 reference file provided in the mothur-wiki pages and filtered the alignment so that all reads overlapped in the same region. The pre.cluster command served to merge reads that were within 2 bp of a more abundant read, and we used UCHIME [22] and Chimera Slayer [20] to identify the chimeric reads. Our sample reads served as a reference for Chimera Slayer.

In QIIME (S2 Text), we followed the 454 Overview Tutorial and Analysis of 18S data available at http://qiime.org/tutorials/index.html# (accessed January–March, 2014). We tested the Denoiser [26] and used pick_otus.py to pick OTUs in de novo mode [46]. The QW filtering step took place under the same parameters as in mothur. For the Chimera Slayer chimera detection, we aligned the reads against the SILVA 111 release reference file (eukarya only, 97% OTUs) using PyNAST [47].

In USEARCH (S3 Text), we followed the UPARSE pipeline available at http://drive5.com/usearch/manual/uparse_pipeline.html (accessed 12 March 2014) and tested the maximum expected error (maxee) and quality score (QS) filtering methods. For the maxee method, we determined the error parameter based on the report given by the fastq_stats command (19% of reads retained). The command discarded reads with > 0.3 expected errors. For the QS filtering method, we used the fastq_truncqual command, which truncated reads at the first position with a quality score 15 or less. We did not search for additional chimeric reads in UPARSE. To obtain globally alignable reads in both the maxee and QS strategies, we truncated the reads to 260 and 200 bp in maxee and QS, respectively.

We generated taxonomic assignment of the 97% OTUs using SILVA database release 111 [48] within the QIIME program package [43] with UCLUST [46] and BLAST [49]. If UCLUST failed to assign the OTU, we used BLAST. In the absence of a taxonomic assignment we treated the OTU as unclassified. For details on the commands used, see S4 Text.

To further validate the results, we manually blasted OTUs affiliated with Metazoa against the NCBI database. For this additional quality control step, we chose Metazoa because the number of Metazoan species in the Baltic Sea is low and well known. In addition, we clustered the chimeric reads identified with UCHIME and Chimera Slayer to 97-% OTUs and blasted the chimeric OTUs assigned with Metazoa to confirm whether the chimeric OTU was an actual chimera.

We used the Shannon index [50] to evaluate differences in alpha-diversity among the bioinformatics strategies, and Whittaker’s beta-diversity to evaluate differences in beta-diversity [51]. We calculated the alpha-diversity values for each sample and the beta-diversity values for the drift ice, pack ice and fast ice.

The number of OTUs and alpha-diversity measures were not normally distributed (Shapiro-Wilk test), so we used the nonparametric Friedman’s repeated measures analysis of variance (the test is used to detect differences in treatments across multiple test attempts) followed by Bonferroni corrected Wilcoxon pairwise comparisons to test whether the different strategies resulted in significantly different numbers of OTUs and alpha-diversity measures.

The raw reads were submitted to the Sequence Read Archive of the European Nucleotide Archive’s (ENA) with accession number PRJEB7625.

## Results

From the 20 samples we obtained 504138 reads (Table 1): 428920 reads for the KOD-Plus- polymerase amplified (16 samples) and 75218 for the Phusion polymerase amplified (4 samples) sets. The average length of the reads was 449 bp (454 bp for the KOD amplified and 423 bp for the Phusion amplified sets). The quality of the Phusion polymerase amplified sample set was poorer as exemplified by the *Eurytemora* classified reads: 45% of the Phusion amplified *Eurytemora* reads were either erroneous or chimeric while only 14% of the KOD amplified *Eurytemora* reads were erroneous or chimeric.

**Table 1:** The basic statistics of the two sequencing data sets.

	Phusion polymerase	KOD-Plus- polymerase
Number of samples	4	16
Number of reads	75218	428920
Number of reverse reads	75218	ca. 204000
Number of forward reads	n/a	ca. 222000
Average read length	423.32	453.85
Average quality score	34.65	35.39
Number of reads classified as *Eurytemora*	420	3500
Percentage of poor quality *Eurytemora* reads	45	14

The two sequencing data sets were generated with Phusion High-Fidelity DNA polymerase and KOD-Plus- ver. 2 polymerase. We classified the raw reads (> 400 bp) with QIIME and investigated the reads classified as *Eurytemora* (Metazoa) in more detail. The forward reads were excluded from the downstream analyses.

After the different quality filtering procedures (Filtering step in Fig 1), the average length of the reads was 200–435 bp, depending on the procedure (Table 2). The read lengths after the UPARSE and mothur strategies were shorter than those after the QIIME strategies because both UPARSE and mothur operate on globally alignable reads (the shortest read in the data set determines the length). The number of unique reads was lower after the denoising strategies (PyroNoise and Denoiser in Fig 1) than after the QW strategies. For example, the resulting numbers of unique reads in QIIME differed by almost two orders of magnitude, ranging from 491 reads in the Denoiser-UCHIME to 43338 reads in the QW-Chimera Slayer strategy. Similarly, the number of unique reads was one third lower after PyroNoise than after QW filtering in mothur, and one third lower after maxee than after QS filtering in UPARSE, revealing substantial differences, depending on the method.

**Table 2:** The number of reads, average length of the reads and the number of chimeras.

	After initial filtering		Chimeras		After all quality control steps	
	Number of reads	Average lenght	Number of chimeras	Chimera percentage	Number of unique reads	Average lenght
UPARSE maxee	44569	466.84	2691^b^	39.80^b^	4069	260.00
UPARSE QS	206969	385.53	3035^b^	17.99^b^	13822	200.00
mothur PyroNoise	197674	277.16				
UCHIME	182622^a^	258.56^a^	27869	15.26	3220	258.44
mothur QW	228095	427.16				
UCHIME	34043^a^	239.01^a^	13476	39.59	8861	239.01
ChimeraSlayer	34043^a^	239.01^a^	13026	38.26	9328	238.84
QIIME Denoiser	121410	439.23				
UCHIME			17842	14.70	491	434.81
QIIME QW	121410	435.07				
UCHIME			20480	16.87	28150	427.92
ChimeraSlayer			0	0	43338	429.68

The number of reads and average length of the reads after the initial quality filtering and after all quality control steps as well as the number of chimeras with the different bioinformatic strategies.

^a^After alignment

^b^Calculated when calling OTUs at the 100% level

The proportion of identified chimeric reads (the chimera removal step in Fig 1) also varied dramatically, depending on the chimera-removal method. The proportion of identified chimeric reads to non-chimeric reads varied among the strategies from zero to 40% (Table 2). UCHIME with mothur found a few hundred more chimeric reads than did Chimera Slayer with mothur. But with QIIME, Chimera Slayer failed and found no chimeric reads. We tried Chimera Slayer using both our reads and the SILVA 111 release as references, but without success. UPARSE has no separate chimera detection step, but reports chimeric reads when calling the OTUs.

The manual blasting of the OTUs classified as Metazoa revealed overestimated numbers of OTUs after all bioinformatic strategies (Table 3). From the OTUs affiliated with Metazoa, 13–93% were erroneous, depending on the strategy; singleton removal, however, improved the quality of the data sets. The QIIME-Denoiser-UCHIME strategy passed the lowest number of erroneous reads, but also missed some high-quality OTUs that other strategies retained. For example, compared to the QIIME-Denoiser-UCHIME strategy, the mothur-PyroNoise-UCHIME strategy resulted in two additional high-quality Metazoan OTUs, but the mothur strategy passed 30 erroneous OTUs more than did the QIIME strategy. In addition, the number of OTUs was higher in mothur than in the QIIME strategies because mothur classified identical reads as different OTUs due to small errors in the multiple alignment mothur used to cluster the reads into OTUs. With QIIME, the QW strategies passed substantially more erroneous reads than did the denoised strategies. None of the strategies tested found all 15 Metazoa OTUs present in the data set (S1 Table). Overall, all strategies passed chimeric reads, but besides chimeric reads UCHIME chimera detection also flagged reads from authentic species as chimeric (Table 3).

**Table 3:** The number of authentic and chimeric Metazoa OTUs.

		Manual blast		UCHIME/ChimeraSlayer removed chimeras	
	N of Metazoa OTUs	Authentic OTUs	Chimeric OTUs	N of Metazoa OTUs	Authentic OTUs
UPARSE					
maxee	11 (5)	6 (4)	5 (1)	n/a	n/a
QS	39 (13)	14 (9)	25 (4)	n/a	n/a
mothur					
PyroNoise					
UCHIME	43 (14)	10 (6)	33 (8)	96	4
QW					
UCHIME	77 (16)	13 (8)	64 (8)	85	1
ChimeraSlayer	91 (17)	14 (9)	77 (8)	73	0
QIIME					
Denoiser					
UCHIME	11 (8)	8 (7)	3 (1)	22	0
No check	32 (23)	8 (7)	24 (16)	n/a	n/a
QW					
UCHIME	36 (23)	9 (7)	27 (16)	113	2
ChimeraSlayer	139 (69)	10 (7)	129 (62)	0	0

The number of Metazoa OTUs generated with the different strategies and removed through chimera detection. We manually checked whether the OTUs originated from actual species or whether they were chimeric or erroneous. Numbers in parentheses are from analyses without singletons.

**Fig 2:**
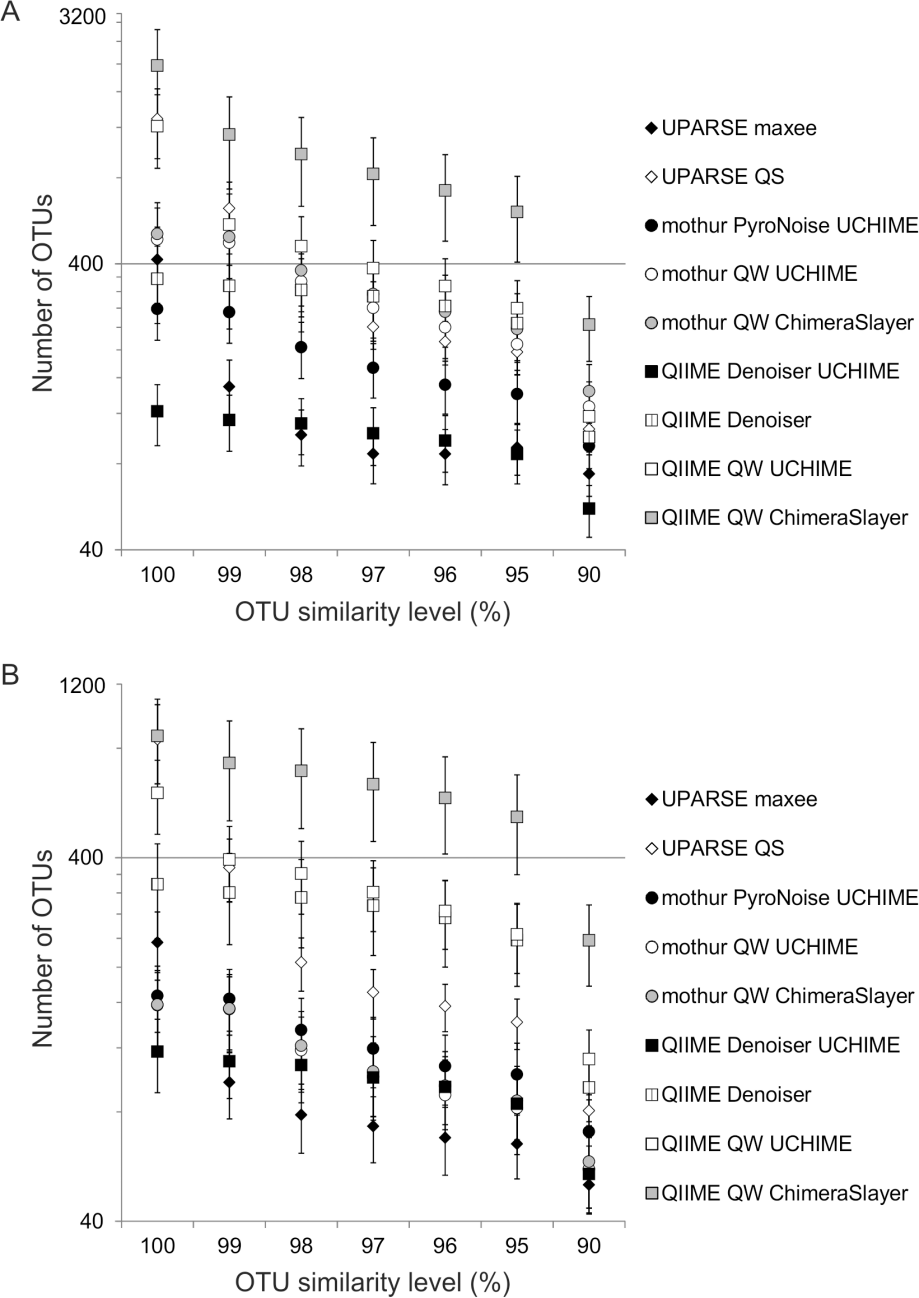
The average number of OTUs at different levels of similarity with different strategies. (A) The average number of OTUs at different levels of similarity with singletons; the y axis is scaled logarithmically. (B) The average number of OTUs at different levels of similarity without singletons; the y axis is scaled logarithmically.

The number of OTUs (following all steps in Fig 1) differed by an order of magnitude, depending on the strategy and quality filtering method (Fig 2). Only the UPARSE-QS strategy yielded the same number of OTUs with singletons as did the mothur-QW-UCHIME and the mothur-QW-Chimera Slayer strategies, and the mothur-QW-UCHIME strategy yielded the same number of OTUs with singletons as the QIIME-Denoiser strategy according to Friedman’s repeated measures analysis of variance (p < 0.001, df = 8 and X^2^ = 945.11) followed by Bonferroni corrected Wilcoxon pairwise comparisons. All strategies yielded significantly different numbers of OTUs after removing singletons (Friedman’s repeated measures analysis of variance, p < 0.001, df = 8, X^2^ = 967.54).

Denoiser produced a three-fold lower average number of OTUs (97%) than did quality filtering: 99 OTUs with the QIIME-Denoiser-UCHIME, and 321 with the QIIME-QW-UCHIME strategies. The effect of PyroNoise in mothur was the opposite: the average number of OTUs (97%) after singleton removal was 119 after PyroNoise and 100 after QW filtering, although the total number of OTUs was lower with PyroNoise (97% OTUs = 456) than QW (97% OTUs = 740). The quality filtering did not succeed as well as PyroNoise did in identifying the reads originating from the same species in the different samples, which yielded more rare OTUs overall, but a smaller number of OTUs per sample with the mothur-QW strategies. The inclusion of singletons overwhelmed this effect with their large numbers: the average number of OTUs (97%) with the mothur-PyroNoise-UCHIME strategy was 173, and with the mothur-QW-UCHIME strategy, 281. Thus, overall, both singleton removal and denoising significantly reduced the number of OTUs (except with PyroNoise and singleton removal in mothur).

**Fig 3:**
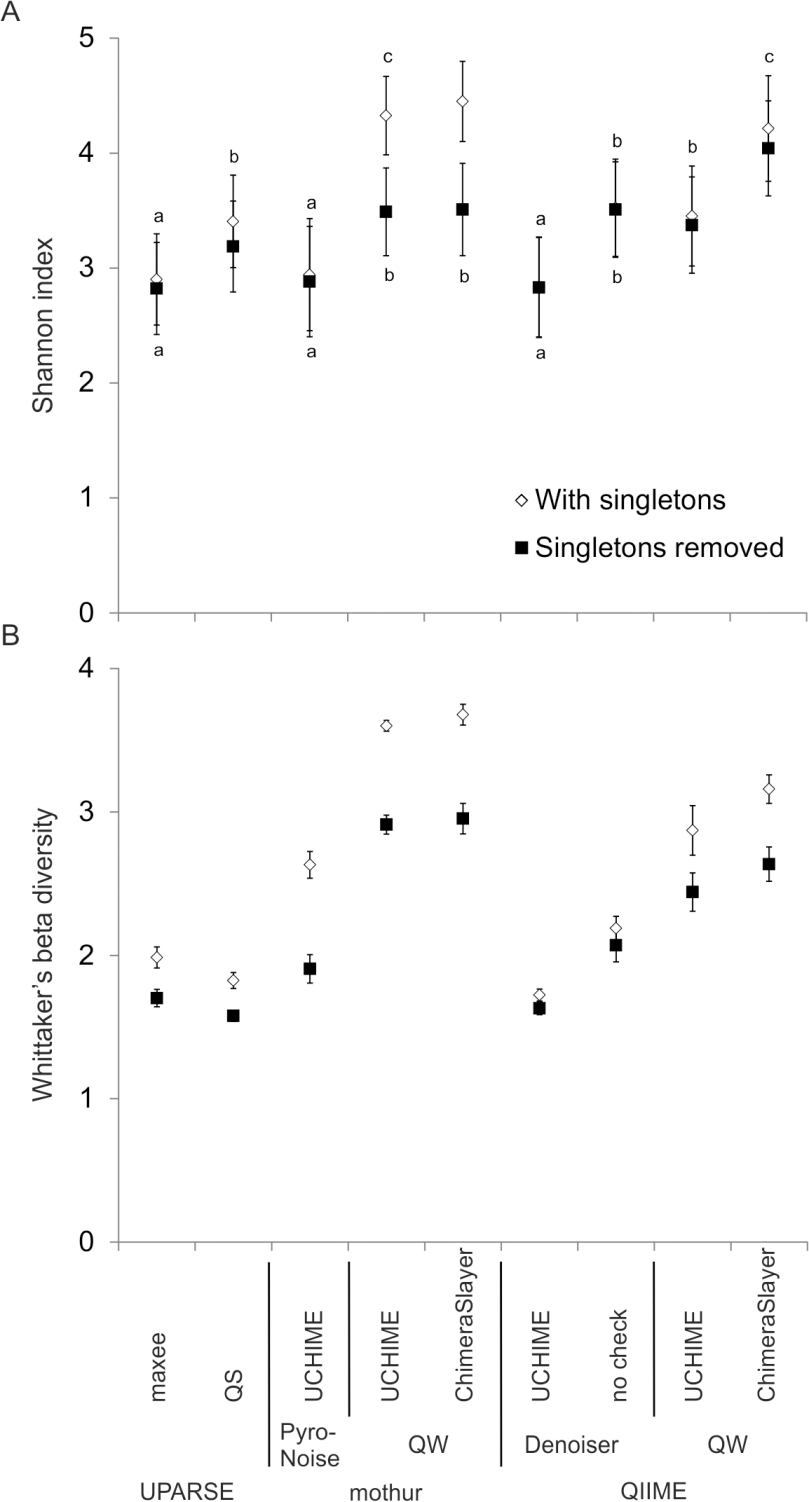
The diversity indices at the 97% OTU level with different bioinformatic strategies. (A) Shannon diversity indices with and without singletons; the small letter on top (with singletons) and below (without singletons) denote similar values from Friedman’s test. (B) Whittaker’s beta-diversity with and without singletons calculated for drift, pack and fast ice.

Alpha-diversity, measured with the Shannon index (Fig 3), grouped the strategies into four groups with the inclusion of singletons (Friedman repeated measures test p < 0.001, df = 8 and X^2^ = 144.21 followed by Bonferroni corrected Wilcoxon pairwise comparisons). With the removal of singletons the strategies were grouped into five groups with equivalent Shannon indices (Friedman repeated measures test p < 0.001, df = 8 and X^2^ = 131.13 followed by Bonferroni corrected Wilcoxon pairwise comparisons). Removing singletons lowered the alpha-diversity measures when using QW strategies, but had no effect on the denoising strategies (Fig 3). Overall, estimating the alpha-diversity attenuated the effect of used strategy but still, significant differences remained among the strategies.

We found no significant difference in Whittaker’s beta-diversity values among the strategies (Fig 3) because their calculation involved only three replicates per strategy (we calculated beta-diversity for drift, pack and fast ice). However, the pattern was clear: singleton removal and denoising reduced beta-diversity.

**Fig 4:**
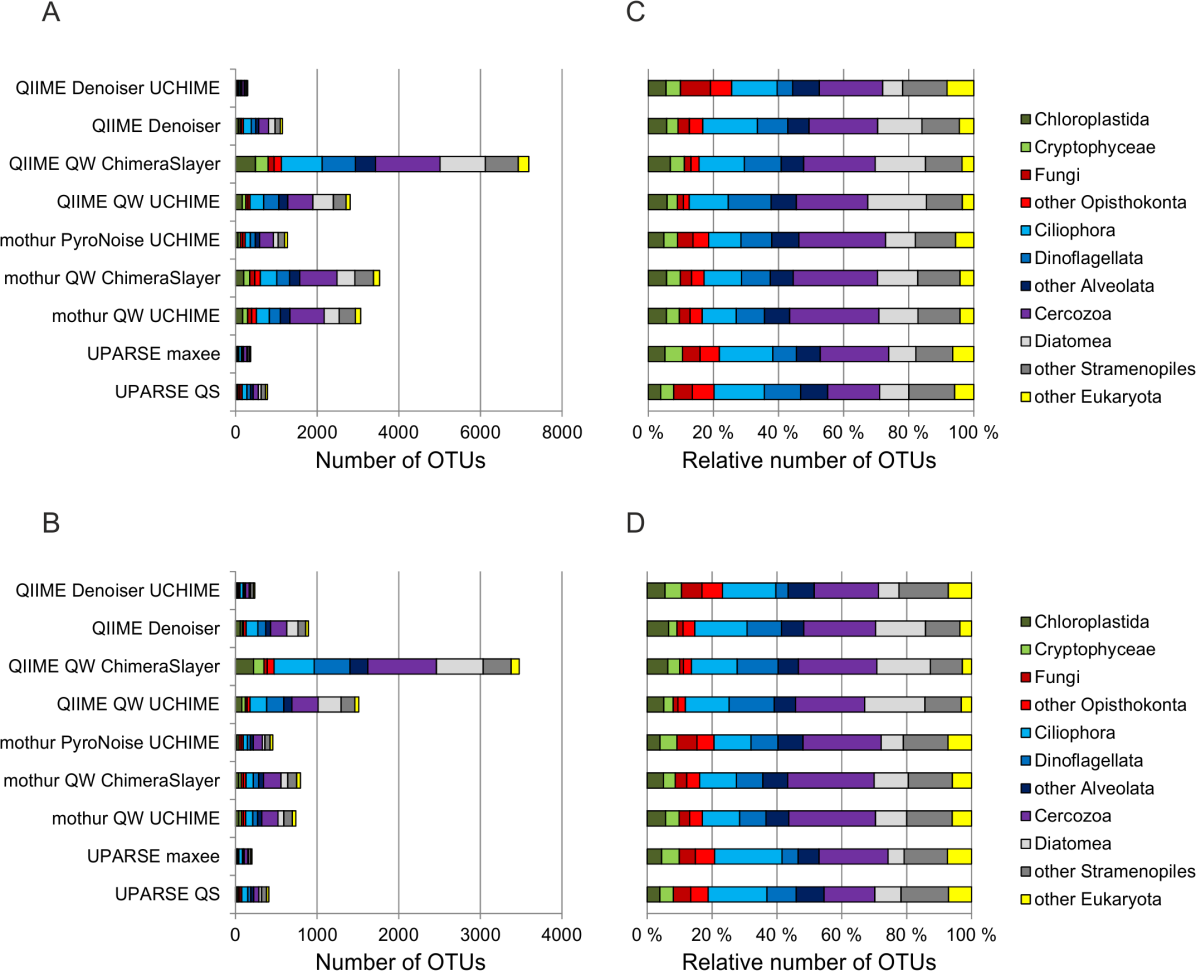
The taxonomic composition of the community revealed with different bioinformatic strategies. (A) The number of OTUs with singletons. B. The number of OTUs without singletons. (C) The relative number of OTUs with singletons. D. The relative number of OTUs without singletons.

In addition to the different numbers of OTUs (Fig 4), the higher-level taxonomic composition of the community (97% OTUs) varied greatly among the different strategies. The most striking difference was in the number of cercozoan OTUs; the number of OTUs affiliated with Cercozoa, which constituted 16–27% of the community, was 78–1580 among different strategies, and after singleton removal, 43–841 (S1 Table). The relative number of cercozoan OTUs was the highest in analyses run in mothur. This was reflected in the proportions of other taxa; the proportion of ciliates, for example, was 10–11% with mothur, but was 12–21% with other strategies. Another example of the variable taxonomic results is the proportion of diatoms, which was 5–9% with UPARSE, mothur-PyroNoise-UCHIME and QIIME-Denoiser-UCHIME strategies, but was 10–19% with the other strategies.

## Discussion

The choice of pre-processing and clustering methods is crucial for the downstream analyses of amplicon read data, as May et al. [28] showed with mock 16S rDNA data. Here, we show with a natural eukaryotic 18S rDNA data set that analyzing the same set of reads with 18 different strategies, can lead to significantly different conclusions.

The different amplicon-read sequencing technologies (e.g., 454 and Illumina) suffer from slightly different issues: the 454-technology is more prone to homopolymer miscounts [25] than is Illumina technology, which has its own base-calling biases [52]. Nevertheless, the results obtained are comparable [34,53]. Thus, our conclusions apply to a broader usage of amplicon-read analyses, although our experimental set-up, in which we used the 454-sequencing technology and three program packages with 18 different amplicon read analysis strategies, is far from exhaustive. Furthermore, the absence of a ground truth complicates analyses of reads derived from natural communities.

### Read Length

Regardless of the sequencing method, reads tend to have more errors towards the end of the read. This is especially distinct in the 454-technology, in which the base quality values of the reads tend to nosedive after approximately 250 bp. This is circumvented to some extent by the demand for globally alignable reads in UPARSE and mothur. This excludes most of the read-end errors [35], but the shortest read in the data set ultimately determines the overall read length. Longer reads provide more information than shorter reads do, and identifying chimeric reads and taxonomic classifications is more difficult with shorter reads. Choosing a longer minimum length in the UPARSE and mothur-QW strategies can lengthen the overall read length of the global alignment but cannot refute the base quality parameters.

In our case, the global alignment resulted in the exclusion of the V7 and V8 regions of the 18S rDNA (in the UPARSE and mothur strategies). For example, QIIME-QW reads were almost twice as long as mothur-QW reads, but because either actual differences or chimeric/erroneous sections were in the V7 and/or V8 region of the QIIME-QW reads, the total and average number of OTUs was higher with the QIIME-QW than with the mothur-QW strategies. Based on our results (Table 3) and the results of Edgar [34], the extra OTUs in the QIIME-QW strategies resulted mainly from chimeric reads. Thus, longer reads do not automatically produce more accurate results.

### To Denoise or Not?

Researchers have proposed several algorithms for removing noise from reads [21,23–26]. This noise consists of, for example, PCR single-base errors, chimeras and errors in sequence reading. Denoising can be not only computationally demanding, but also unnecessary [34]. The main concerns of denoising include the removal of actual species [27] and changes in taxonomic distribution [38]. However, adding a denoising step improves the OTU clustering, as most erroneous reads closely resemble their parental reads, and denoising alters the erroneous reads to such extent that they are part of their parental reads [28]. Several studies recommend using denoising, which results in more accurate numbers of OTUs in mock analyses [14,23,25,26,28,38]. Also, using at least two runs under different emulsion PCR and sequencing conditions is beneficial for separating noise from biological variation [39].

Gaspar and Thomas [35] thoroughly examined different denoising approaches, and found that PyroNoise [21] implemented in AmpliconNoise substantially changed the reads. Because it picked the longest read as the representative of each cluster, it added bases to the 3’ ends of the shorter reads that were often dissimilar from what truncation had previously removed. The Denoiser algorithm [26] implemented in QIIME caused even more changes than did PyroNoise, and most of these changes were substitutions. The PyroNoise in mothur made markedly fewer changes than in AmpliconNoise and Denoiser in QIIME owing to its strict filtering criteria. However, due to these criteria the reads were shorter than in AmpliconNoise and QIIME. This is why Gaspar and Thomas [35] recommend being aware of and examining how the denoising process transforms the reads.

None of the bioinformatic strategies tested found all 15 Metazoa OTUs present in the data set (S1 Table). Denoised strategies missed more actual species than did QW strategies, but the QW strategy results included substantially more chimeric reads than did the denoised strategy results. For example, the QIIME-Denoiser-UCHIME resulted in almost an order of magnitude fewer chimeric OTUs than did the QIIME-QW-UCHIME strategy. The PyroNoise denoising shortened the reads substantially from 449 bp to 277 bp, while Denoiser denoising shortened the reads by only 10 bp. The Denoiser altered reads to such that the QIIME-Denoiser-UCHIME strategy yielded 13% fewer chimeric reads than did the QIIME-QW-UCHIME strategy (Table 2). Moreover, Denoiser lost at least three actual Metazoa OTUs present in the OTUs or UCHIME/Chimera Slayer-removed chimeric reads of the QIIME-QW strategies (Table 3, S1 Table). PyroNoise denoising implemented in mothur lost no OTUs. In accordance with the results of Gaspar and Thomas [35], denoising had more pronounced effect on the number of OTUs in QIIME than in mothur because the QIIME-QW strategy passed more chimeric reads than did the mothur-QW strategy.

### Chimera Detection

A compromise exists between the sensitivity and specificity of chimera detection methods: improving sensitivity decreases specificity. Although UCHIME and Chimera Slayer are the most sensitive methods available [22], they perform far from ideally when used in default (Table 3), consequently, one should evaluate all chimera-detection results carefully. Chimeric reads that pass chimera detection are the main reason for inflated estimates of diversity [14], but detection methods also flag actual OTUs chimeric as well. False positives and negatives may cause spurious inferences of differences between populations (see the discussion in the To Denoise or Not? section).

Several authors [12,13,27,34,54,55] recommend singleton (or rare OTU) removal as the easiest method for chimera removal. This approach is very effective, but can evidently be used only when the investigator is uninterested in the rare biosphere [39]. For example, in our data set, OTUs affiliated with Amoebozoa, Centrohelida, Hypchytriales and Peronosporomycetes were seldom detected as a consequence of singleton removal (S1 Table).

In our case, the failure of Chimera Slayer in QIIME may be a result of our simplified approach. We did not aim to customize the strategies too much as the pipelines are intended for non-expert end users in bioinformatics (microbiologists with ecological question settings). In addition, we implemented chimera detection in the same phase for all strategies: between initial quality control or denoising and OTU calling (S1 and S2 Text). The QIIME development team suggests (http://qiime.org/scripts/identify_chimeric_seqs.html) using Chimera Slayer after picking the representative set. However, with that approach, Chimera Slayer failed as well. May et al. [28] recommend chimera detection before denoising in order to simplify the denoising step, which is advantageous when computational time and power are limited. As a better way to remove most of the chimeras, May et al. [28] also suggest running UCHIME against both one’s own sequences and a curated reference sequence set and then combining the results.

### Diversity Estimates

OTU richness among the strategies differed widely (Fig 2). Bachy et al. [37] showed with tintinnid ciliates that the multiple alignment needed to assign the reads into OTUs with mothur can include small errors, which can lead to a 10- or even 100-fold overestimation of OTUs at high levels of similarity (99%). This effect is somewhat attenuated at lower levels of similarity, but still visible in our results. This can be corrected with an additional alignment step.

The primary aim of most environmental sequencing studies is to compare the diversity of a set of samples [56–58]. To that end, one can calculate several alpha- and beta-diversity measures [59]. These measures variably take into account the presence and abundance of OTUs. For simplicity, we chose two traditional measures of diversity to compare the performance of the bioinformatic strategies: the Shannon index [50] and Whittaker’s beta-diversity [51].

The Shannon index attenuated differences among the strategies and found clear groupings. Shannon indices were lower with denoised strategies than with QW strategies because of the lower number of spurious OTUs and the more even abundance of OTUs in denoised strategies. Thus, for the purpose of estimating the eukaryotic diversity of examined environments, the choice of bioinformatic pipeline is less about choosing the program package than about choosing between denoising and no denoising and even more about choosing the diversity estimate to use [59,60].

The huge variability in copy numbers in the 18S rDNA of the different eukaryotic lineages [29–31] hampers ecological interpretation of abundance-based alpha-diversity measures. The read abundance does not reflect the biomass or cell number of eukaryotes [38]. Read abundance data and abundance-based estimates of diversity should therefore be used only in a strict sense to compare different samples without further ecological interpretations.

### Effects of Bioinformatic Strategies on Community Composition

We found distinctive differences among the strategies in the community composition results (Fig 4), differences so striking that they led to contradictory conclusions about the composition of the community. Interestingly, the effect was clearly taxon-specific, with the highest variation occurring in Cercozoa (S1 Table). Behnke et al. [13] studied in detail the 454-sequencing errors in V4 and V9 of ciliates, diatoms and Rhizaria (which includes Cercozoa). They found that the GS-FLX Titanium error rates in V9 for Rhizaria were twice as high as for ciliates, and four times higher than for diatoms. Plausible reasons for these taxon-specific differences include varying numbers of long homopolymer stretches, variable secondary structure formation and the presence of additional hairpins and branched structures [13,61,62]. Taxon specificity has distinct implications for the analyses. Firstly, defining a universal OTU similarity level for all eukaryotes is impossible. Secondly, the results gained with, for example, ciliates as models for amplicon read analyses [27,37] may not hold for other eukaryotic taxa. Both issues are solvable through further studies on taxon-specific differences in sequencing technologies and on structures of ribosomal RNA.

## Supporting Information

S1 Table The number of OTUs in different taxonomic groups. (DOCX)

S1 Text The detailed commands used in mothur. (TXT)

S2 Text The detailed commands used in QIIME. (TXT)

S3 Text The detailed commands used in USEARCH. (TXT)

S4 Text The detailed commands used for taxonomic assignment. (TXT)
