# Supplementary material for: Bioinformatic Amplicon Read Processing Strategies Strongly Affect Eukaryotic Diversity and the Taxonomic Composition of Communities
Source: PLoS One. 2015 Jun 5;10(6):e0130035. doi: 10.1371/journal.pone.0130035 (PMC4457843; doi:10.1371/journal.pone.0130035)
Supplement: S1 Table — (DOCX) [file pone.0130035.s001.docx]

S1 Table. The number of OTUs in different taxonomic groups. Numbers in parenthesis are from analyses without singletons. The presence of good quality Metazoa OTUs are listed based on the manual blasting (grey background).

|  | | | | | | | |  | | |  | |  |  | | |  | |  |  | | |  | |  |
| --- | --- | --- | --- | --- | --- | --- | --- | --- | --- | --- | --- | --- | --- | --- | --- | --- | --- | --- | --- | --- | --- | --- | --- | --- | --- |
|  | | | | | | | |  |  |  |  |  |  |  |  |  |  |  |  |  |  |  |  |  |  |
|  |  |  |  | |  |  |  | |  |  | |  | | |  |  | |  | | |  |  | |  | |
|  |  |  | UPARSE | | |  | mothur | | | | | | | |  | QIIME | | | | | | | | | |
|  |  |  | maxee | QS | |  | PyroNoise | |  | QW | | | | |  | Denoiser | | | | |  | QW | | | |
|  |  |  |  |  | |  | UCHIME | |  | UCHIME | | Chimera Slayer | | |  | UCHIME | | No check | | |  | UCHIME | | Chimera Slayer | |
| uncultured Eukaryota | | |  | 6 (3) | |  | 13 (3) | |  | 17 (4) | | 21 (3) | | |  | 1 (1) | | 4 (1) | | |  | 12 (7) | | 38 (9) | |
| Amoebozoa | |  |  | 1 (1) | |  |  | |  | 3 (0) | | 3 (0) | | |  | 1 (0) | | 1 (0) | | |  | 1 (0) | | 1 (0) | |
| Chloroplastida | |  | 19 (9) | 30 (16) | |  | 61 (18) | |  | 171 (42) | | 199 (40) | | |  | 16 (13) | | 65 (59) | | |  | 162 (77) | | 487 (221) | |
| Glaucophyta | |  |  |  | |  |  | |  | 1 (0) | |  | | |  |  | | 1 (0) | | |  | 1 (0) | | 6 (1) | |
| Centrohelida | |  | 1 (1) | 3 (1) | |  | 2 (0) | |  | 1 (0) | | 1 (0) | | |  |  | |  | | |  |  | |  | |
| Cryptophyceae | |  | 20 (11) | 31 (17) | |  | 53 (24) | |  | 122 (31) | | 149 (29) | | |  | 13 (12) | | 40 (23) | | |  | 89 (44) | | 311 (128) | |
| Discoba | |  | 2 (0) | 4 (3) | |  | 1 (1) | |  | 4 (3) | | 5 (3) | | |  | 4 (3) | | 4 (3) | | |  | 4 (3) | | 4 (3) | |
| Haptophyta | |  | 7 (6) | 14 (10) | |  | 18 (12) | |  | 38 (14) | | 43 (15) | | |  | 8 (7) | | 19 (15) | | |  | 40 (19) | | 103 (44) | |
| Apusomonadidae | |  |  | 1 (1) | |  | 1 (1) | |  | 1 (0) | | 1 (0) | | |  | 2 (0) | | 2 (0) | | |  | 2 (0) | | 2 (0) | |
| Telonema | |  | 2 (2) | 3 (2) | |  | 7 (5) | |  | 12 (5) | | 15 (7) | | |  | 3 (2) | | 4 (2) | | |  | 6 (3) | | 12 (4) | |
| Kathablepharidae | |  | 2 (1) | 4 (3) | |  | 7 (3) | |  | 10 (3) | | 9 (3) | | |  | 3 (2) | | 3 (2) | | |  | 3 (2) | | 8 (2) | |
| Fungi | |  | 20 (10) | 45 (22) | |  | 61 (28) | |  | 100 (24) | | 121 (28) | | |  | 27 (15) | | 40 (17) | | |  | 52 (22) | | 146 (40) | |
| uncultured Opisthokonta | | | 1 (0) | 1 (0) | |  | 3 (0) | |  | 5 (0) | | 6 (0) | | |  |  | |  | | |  |  | | 1 (0) | |
| uncultured Holozoa | | |  | 1 (1) | |  | 2 (2) | |  | 4 (3) | | 7 (3) | | |  | 1 (1) | | 1 (1) | | |  | 2 (2) | | 5 (2) | |
| Choanomonada | |  | 9 (6) | 10 (7) | |  | 12 (7) | |  | 26 (9) | | 29 (11) | | |  | 7 (6) | | 13 (8) | | |  | 11 (9) | | 28 (12) | |
| Corallochytrium | |  |  | 1 (1) | |  | 1 (1) | |  | 1 (1) | | 1 (1) | | |  |  | |  | | |  | 1 (1) | | 1 (1) | |
| Filasterea | |  |  |  | |  |  | |  |  | |  | | |  |  | |  | | |  |  | | 1 (0) | |
| Ichthyosporea | |  | 1 (1) |  | |  |  | |  | 2 (0) | | 3 (0) | | |  |  | | 1 (0) | | |  |  | | 3 (1) | |
| Metazoa | |  | 11 (5) | 39 (13) | |  | 43 (14) | |  | 77 (16) | | 91 (17) | | |  | 11 (8) | | 32 (23) | | |  | 36 (23) | | 140 (69) | |
|  | Crustacea | *Eurytemora affinis* | x (x) | x (x) | |  | x (x) | |  | x (x) | | x (x) | | |  | x (x) | | x (x) | | |  | x (x) | | x (x) | |
|  |  | *Limnocalanus macrurus* | | x (x) | |  |  | |  |  | | x (x) | | |  | x (x) | | x (x) | | |  | x (x) | | x (x) | |
|  |  | *Temora* sp. |  | x | |  | x | |  | x (x) | | x (x) | | |  |  | |  | | |  |  | | x | |
|  |  | Calanidae sp. 1 |  | x | |  |  | |  | x | | x | | |  |  | |  | | |  |  | |  | |
|  |  | Calanidae sp. 2 |  |  | |  |  | |  | x (x) | | x (x) | | |  |  | |  | | |  |  | |  | |
|  |  | *Balanus* sp. |  | x (x) | |  |  | |  |  | |  | | |  | x (x) | | x (x) | | |  | x (x) | | x (x) | |
|  | Insecta | *Lepisma* sp. | x (x) | x (x) | |  | x (x) | |  | x (x) | | x (x) | | |  | x (x) | | x (x) | | |  | x (x) | | x (x) | |
|  | Rotifera | *Synchaeta* sp. | x (x) | x (x) | |  | x (x) | |  | x (x) | | x (x) | | |  | x (x) | | x (x) | | |  | x (x) | | x (x) | |
|  |  | *Keratella* sp. |  | x | |  |  | |  | x | | x | | |  |  | |  | | |  |  | |  | |
|  | Annelida | *Marenzelleria* sp. | x (x) | x (x) | |  | x (x) | |  | x (x) | | x (x) | | |  | x (x) | | x (x) | | |  | x (x) | | x (x) | |
|  | Mollusca | *Macoma balthica* |  | x (x) | |  | x (x) | |  | x (x) | | x (x) | | |  |  | |  | | |  |  | |  | |
|  | Priapulida | *Halicryptus spinulosus* |  | x | |  | x | |  | x | | x | | |  | x | | x | | |  | x | | x | |
|  | Ctenophora | *Mertensia ovum* |  | x | |  | x | |  | x | | x | | |  |  | |  | | |  |  | |  | |
|  | Chordata | *Salmo* sp. | x | x (x) | |  | x | |  | x | | x | | |  |  | |  | | |  | x | | x | |
|  |  | Mammalia sp. | x | x (x) | |  | x (x) | |  | x (x) | | x (x) | | |  | x (x) | | x (x) | | |  | x (x) | | x (x) | |
| Picozoa | |  | 1 (1) |  | |  | 2 (1) | |  | 3 (1) | | 3 (1) | | |  | 1 (1) | | 3 (2) | | |  | 2 (1) | | 10 (4) | |
| uncultured SAR | |  | 7 (3) | 8 (4) | |  | 19 (6) | |  | 35 (13) | | 43 (14) | | |  |  | | 9 (6) | | |  | 25 (10) | | 73 (30) | |
| uncultured Alveolata | | | 17 (9) | 44 (23) | |  | 79 (23) | |  | 194 (43) | | 207 (53) | | |  | 15 (12) | | 65 (53) | | |  | 199 (88) | | 462 (203) | |
| Apicomplexa | |  |  |  | |  | 1 (0) | |  | 1 (0) | | 1 (0) | | |  | 1 (0) | | 1 (0) | | |  | 1 (0) | | 1 (0) | |
| Ciliophora | |  | 61 (42) | 121 (74) | |  | 126 (52) | |  | 320 (85) | | 405 (90) | | |  | 41 (39) | | 193 (144) | | |  | 337 (204) | | 997 (491) | |
| Dinoflagellata | |  | 27 (10) | 87 (37) | |  | 120 (38) | |  | 266 (60) | | 312 (65) | | |  | 14 (9) | | 107 (95) | | |  | 368 (210) | | 814 (439) | |
| Protalveolata | |  | 10 (4) | 21 (12) | |  | 26 (12) | |  | 41 (9) | | 40 (8) | | |  | 8 (7) | | 9 (8) | | |  | 18 (11) | | 33 (14) | |
| uncultured Rhizaria | | |  |  | |  |  | |  | 3 (0) | | 4 (0) | | |  |  | |  | | |  | 2 (2) | | 2 (1) | |
| Cercozoa | |  | 78 (43) | 125 (64) | |  | 339 (110) | |  | 840 (198) | | 915 (212) | | |  | 57 (47) | | 242 (198) | | |  | 616 (322) | | 1580 (841) | |
| uncultured Stramenopiles | | | 4 (1) | 12 (7) | |  | 11 (4) | |  | 36 (11) | | 41 (8) | | |  | 3 (2) | | 10 (6) | | |  | 21 (11) | | 55 (23) | |
| Bicosoecida | |  |  | 3 (2) | |  | 4 (0) | |  | 3 (0) | | 5 (0) | | |  | 1 (1) | | 1 (1) | | |  | 1 (1) | | 2 (1) | |
| Bolidomonas | |  | 3 (2) | 7 (4) | |  | 10 (3) | |  | 38 (9) | | 38 (8) | | |  | 4 (3) | | 8 (6) | | |  | 39 (23) | | 83 (38) | |
| Chrysophyceae | |  | 20 (11) | 50 (26) | |  | 60 (27) | |  | 173 (47) | | 207 (55) | | |  | 16 (16) | | 68 (52) | | |  | 157 (93) | | 398 (189) | |
| Diatomea | |  | 31 (10) | 70 (33) | |  | 116 (31) | |  | 369 (71) | | 436 (84) | | |  | 18 (15) | | 156 (137) | | |  | 505 (281) | | 1110 (573) | |
| Dictyochophyceae | |  | 4 (4) | 8 (6) | |  | 15 (8) | |  | 26 (13) | | 33 (13) | | |  | 4 (4) | | 9 (8) | | |  | 19 (9) | | 51 (17) | |
| Eustigmatales | |  | 1 (1) | 4 (2) | |  | 4 (1) | |  | 16 (3) | | 16 (3) | | |  | 1 (1) | | 2 (2) | | |  | 11 (5) | | 32 (8) | |
| Pirsonia | |  |  |  | |  | 1 (1) | |  |  | |  | | |  | 1 (1) | | 1 (1) | | |  | 1 (1) | | 4 (1) | |
| Labyrinthulomycetes | | | 4 (3) | 9 (7) | |  | 19 (9) | |  | 31 (7) | | 34 (8) | | |  | 5 (4) | | 14 (4) | | |  | 10 (5) | | 45 (13) | |
| MAST-1 | |  | 2 (1) | 4 (2) | |  | 8 (3) | |  | 25 (6) | | 32 (8) | | |  | 1 (1) | | 12 (10) | | |  | 24 (11) | | 76 (34) | |
| MAST-12 | |  |  | 3 (0) | |  | 1 (1) | |  | 4 (0) | | 4 (0) | | |  |  | |  | | |  |  | |  | |
| MAST-6 | |  | 1 (1) |  | |  | 3 (1) | |  | 2 (2) | | 1 (1) | | |  | 1 (1) | | 1 (1) | | |  | 1 (1) | | 5 (2) | |
| Opalinata | |  |  | 1 (0) | |  |  | |  |  | | 1 (0) | | |  |  | |  | | |  |  | |  | |
| Pelagophyceae | |  | 1 (1) | 2 (1) | |  | 5 (1) | |  | 22 (2) | | 21 (1) | | |  | 2 (1) | | 4 (2) | | |  | 5 (2) | | 28 (9) | |
| Peronosporomycetes | | |  | 4 (1) | |  | 6 (1) | |  | 6 (0) | | 7 (0) | | |  |  | |  | | |  | 4 (0) | | 4 (0) | |
| Phaeothamniophyceae | | |  |  | |  |  | |  |  | | 1 (0) | | |  |  | |  | | |  | 1 (0) | | 1 (0) | |
| Raphidophyceae | |  | 1 (1) | 3 (2) | |  | 8 (1) | |  | 12 (3) | | 14 (2) | | |  | 1 (1) | | 1 (1) | | |  | 5 (3) | | 10 (3) | |
| Synurales | |  | 1 (1) |  | |  | 2 (2) | |  | 2 (1) | | 3 (1) | | |  |  | | 1 (1) | | |  | 9 (4) | | 11 (4) | |
| Xanthophyceae | |  |  |  | |  |  | |  |  | |  | | |  |  | |  | | |  | 1 (0) | | 1 (0) | |
| Unclassified | |  | 2 (1) | 2 (1) | |  | 1 (1) | |  | 1 (1) | | 1 (1) | | |  | 1 (1) | | 1 (1) | | |  | 1 (1) | | 1 (1) | |
